# On-device query intent prediction with lightweight LLMs to support ubiquitous conversations

**DOI:** 10.1038/s41598-024-63380-6

**Published:** 2024-06-03

**Authors:** Mateusz Dubiel, Yasmine Barghouti, Kristina Kudryavtseva, Luis A. Leiva

**Affiliations:** University of Luxembourg, 4365 Esch-sur-Alzette, Luxembourg

**Keywords:** Conversational Agents, Design, Information retrieval, Graphical user nterfaces, Computational science, Computer science, Information technology

## Abstract

Conversational Agents (CAs) have made their way to providing interactive assistance to users. However, the current dialogue modelling techniques for CAs are predominantly based on hard-coded rules and rigid interaction flows, which negatively affects their flexibility and scalability. Large Language Models (LLMs) can be used as an alternative, but unfortunately they do not always provide good levels of privacy protection for end-users since most of them are running on cloud services. To address these problems, we leverage the potential of transfer learning and study how to best fine-tune *lightweight* pre-trained LLMs to predict the intent of user queries. Importantly, our LLMs allow for on-device deployment, making them suitable for personalised, ubiquitous, and privacy-preserving scenarios. Our experiments suggest that RoBERTa and XLNet offer the best trade-off considering these constraints. We also show that, after fine-tuning, these models perform on par with ChatGPT. We also discuss the implications of this research for relevant stakeholders, including researchers and practitioners. Taken together, this paper provides insights into LLM suitability for on-device CAs and highlights the middle ground between LLM performance and memory footprint while also considering privacy implications.

## Introduction

When using cloud-based communication platforms, users often lose control over their privacy, as their data is processed by (and ends up being stored on) third-party servers, which may also be used for further training by service providers. Moreover, as indicated by prior work, users’ privacy intentions are often not in sync with their behaviour, which may lead to users unwittingly disclosing sensitive information^1,2^. This issue is pertinent when it comes to interaction with systems that are designed to mimic human-like interaction such as Conversational Agents (CAs)^3,4^, especially on mobile devices that can be considered as ‘intimate’ objects that users rarely part with^5^.

Nowadays, CAs are becoming increasingly ubiquitous. They come in many shapes and forms, such as digital assistants on smartphones (e.g., *Apple Siri*, *Google Assistant*, *Samsung Bixby*), stand-alone devices (e.g., *Amazon Echo Show*, *Google Nest*, and *Tencent Tingting*), or automotive systems (e.g., *BMW Intelligent Personal Assistant*, *Cerence Automotive Platform*), just to name a few. Popular areas of CA applications include e.g. health and well-being^6–8^, tutoring^9–11^, and productivity^6,12,13^.

As hinted before, CAs that are running on a cloud service do not always provide good levels of privacy protection, since users have no guarantee that their voice or text commands will be safely handled there^14^. While it has been demonstrated that traditional CAs can run completely offline, even on low-resource devices such as a RaspberryPi^14^, this approach does not scale well. Specifically, traditional approaches for developing CAs involve use of predefined slot-filling mechanisms^15,16^ and rigid interaction flows^17^, consequently hindering flexibility and scalability to new tasks or domains^18^. For example, a user’s utterance “*Show my savings account balance*” indicates a “show-balance” intent with an “account-type” slot. Overall, training a slot-filling system is challenging, as it requires considerable manual encoding of multiple variations of user utterances for each slot and each intent. In fact, this approach is considered deprecated nowadays^19,20^, only suitable for very simple situations such as those were users have to choose between some given options; cf. the customer service of a call centre.

Machine Learning (ML) is an alternative approach that allows for the CA’s behaviour to be learned from data, without the need to program it explicitly, which makes it a more scalable and generalisable. More concretely, transfer learning has recently emerged as a de-facto ML method in Natural Language Processing (NLP), where pre-trained Large Language Models (LLMs) are adapted to new tasks by fine-tuning their hyperparameters on a small but representative dataset. However, fine-tuning most modern LLMs (e.g. PaLM, LLaMA, or the GPT family) is out of reach to many researchers due to high computational requirements^21^ and associated high monetary costs^22^. Therefore, quite often, the only option for many researchers and practitioners is to rely on a cloud-based service that provides an interface to those LLMs, thereby compromising the user’s privacy, especially for CAs that operate with sensitive information^23^ or are designed to encourage information disclosure^24^.

We should note that, in this paper, by ‘model’ we refer to ‘computational model’, i.e., a data-driven structure that is trained (through examples) to map inputs and outputs. Therefore, a computational model is both structure and data. As noted, without data, no model pre-training is possible. Furthermore, there is ample evidence that supports the claim that high-quality data makes better models, and not the other way around^25,26^. We elaborate more on this observation in the ‘Implications’ section at the end of this paper.

**Figure 1:**
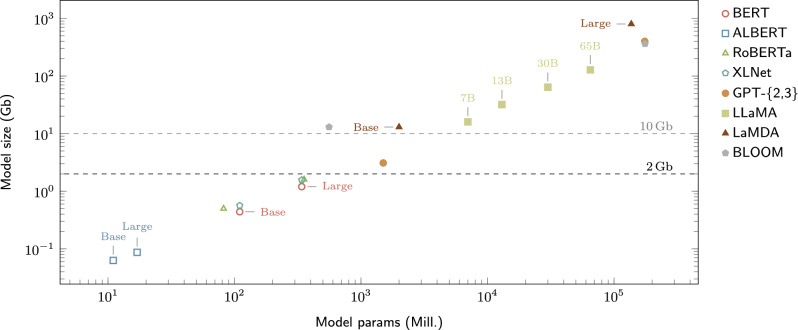
Overview of some popular LLMs. Axes are in logarithmic scale to ease the visualization (almost a linear relationship between model size and model parameters). Each model has different variants available, such as Base and Large (see the plot annotations). As discussed in the next section, we set 2 Gb as the upper-bound for LLM size so that it can be deployed on commodity mobile and ubiquitous devices (see ‘Models’ section for more details). Therefore, in this paper, only the models below such an upper-bound are considered *lightweight*.

To bridge the gap between user’s privacy and scalability of LLM-based CAs, we investigate transfer learning on *lightweight* LLMs that can be deployed for on-device inference tasks, a fundamental pre-requisite for mobile and ubiquitous systems; see Fig. 1. Specifically, we look at predicting four query intent surrogates, described in ‘Methods’ section. Intent surrogates are crucial to understand the context of interactive conversations, as they determine the efficiency of a CA when it comes to correctly interpreting user’s input and successfully addressing it. Our investigation taps into the “Conversations with GUIs” dataset^27^, as it provides an interesting testbed for mobile systems, as explained in ‘Materials’ section.

GUI datasets such as Rico^28^, Enrico^29^, VINS^30^, or WebUI^31^ can be useful during the early stages of design and development of applications by providing inspiration and insights into various app features. While such datasets contain rich information regarding GUI properties and relevant technical specification, querying them may require using developer expertise or sophisticated JSON-based APIs^32^, making them inaccessible for users without programming experience. In order to address this problem, Todi et al.^27^ proposed the use of a conversation modality to support users navigate complex GUI datasets using natural language. In this paper, we further explore this concept with a series of lightweight LLMs suitable for *on-device* NLP tasks. It should be noted that while prompt engineering allows for a more efficient use of LLMs through developing and optimising instructions to guide the model^33^, it is not supported by the lightweight LLMs that we explore in this paper. However, for comparative purposes we also assess the performance of a larger, state-of-the-art LLM, (ChatGPT) which is fine-tuned with prompt engineering.

While LLMs are becoming increasingly ubiquitous they are susceptible to data leakage^34^, posing a treat to end-users’ privacy. In order to explore alternatives to regular LLMs that rely on external cloud services for deployment, here we investigate the performance of lightweight LLMs on tasks that involve predicting query intents while running on commodity mobile devices such as smartphones and tablets that can be used ‘on the go’. Following Stal et al.^35^ we define mobile device as, “a portable, wireless computing device, possible to carry without additional equipment and small enough to be used while held in the hand”. We formulate the following research questions:

- **RQ1** concerns the performance of the models on intent prediction tasks:
  - **RQ1a:** *Which pre-trained models achieve the best performance after fine-tuning for predicting query intents in different tasks?*
  - **RQ1b:** *Is there a model that performs best in all of the tasks?*
- **RQ2** concerns the relationship between model performance and fine-tuning time:
  - **RQ2a:** *What is the minimum number of fine-tuning epochs for each pre-trained model?*
  - **RQ2b:** *What is the optimal number of fine-tuned epochs for each model to achieve the best performance?*

By addressing the above research questions, our work makes the following contributions:

- We provide insights into adequacy of lightweight LLMs for on-device NLP tasks and their fit for specific types of user queries in the context of GUI conversations. We also conduct additional experiments on other datasets, for completeness.
- We shed light on the performance versus privacy trade-off and demonstrate the feasibility of deploying LLMs-based CAs on mobile and ubiquitous devices. We show that lightweight LLMs require more fine-tuning epochs than previously assumed to reach their peak performance.
- We discuss the implications of our research for different types of stakeholders, including researchers, developers, designers, and end-users. While ChatGPT excels at zero-shot classification tasks, lightweight LLMs achieve similar performance (sometimes even better) after fine-tuning.

Overall, this work makes an empirical contribution to mobile and ubiquitous systems that need to effectively balance performance and memory footprint, while also considering privacy implications for end-users. More specifically, we make the selection of specific pre-trained models more informed, and help to avoid a trial-and-error selection approach when applying lightweight LLMs to develop CAs. The main premise of this paper is that LLMs should be fine-tuned on commodity hardware, without the need to access High Performance Computing facilities or a cloud service provider.

## Background and related work

We discuss the relevance of CAs to overcome the challenge of capturing users’ information needs, also know as the *semantic gap*, and explain how transfer learning is transformative to this challenge. We also discuss current privacy and sustainability issues of LLMs that may have an important impact on their widespread use.

### Conversational Agents for GUI interactions

The ‘CA’ term is an umbrella term which includes two main types of automated dialogue systems, namely (1) task-oriented agents designed to accomplish a specific goal (e.g., booking a flight) and (2) non-task-oriented agents^17^. In this paper, we focus on the former sense, considering an agent whose goal is to support a user in the task of GUI dataset exploration through natural language.

We can find recent examples of CAs that have been applied to GUI design and layout tasks such as: sketching^36^, creating task shortcuts to UI screens in apps^37^, and creating low-fidelity UI mock-ups from natural language phrases^38^. Most relevant to our investigation, however, is the work of Todi et al.^27^ who presented a CA prototype to explore a large body of visual designs from their “Conversations with GUIs” dataset. The prototype provided answers to users’ questions in the form of text, numbers, GUIs, or a part of their design. For example, users could issue queries such as “*Show me examples of search bar designs*” or “*When was the app last updated?*” to find information that can help them satisfy their search needs or provide a useful point of reference.

Recently, the topic of resolving user information needs through conversation has been receiving increasing interest, leading to development of several CA-based interactive systems^39–41^. For example, Jahanbakhsh et al.^40^ built a human-in-the-loop AI question answering system to assist users with business documents. The system was well-aligned with the needs of actual users, as their questions were collected in-situ while users were working on their documents naturally (i.e., conducting their everyday work tasks). In another study, Wang et al.^42^ investigated the feasibility of enabling a versatile conversational interactions with mobile interfaces using an LLM. While they designed prompting techniques to adapt an LLM to mobile UIs, they barely explored informational queries for single-UI interactions. In our work, we also explore navigational queries and extend the study beyond GUIs to (i) full mobile applications and (ii) datasets.

### Sentiment analysis with LLMs

Another research area that is relevant to our work is Sentiment Analysis, an NLP technique whose goal is to examine the emotional tone of an utterance or piece of text. Varia et al.^43^ proposed an unified framework for solving Aspect-based Sentiment Analysis (ABSA). ABSA is a sentiment analysis task that involves four elements from user-generated texts: aspect term, aspect category, opinion term, and sentiment polarity. Varia et al. fine-tuned a T5 model with instructional prompts in a multi-task learning fashion covering all the sub-tasks, as well as the entire quadruple prediction task. They showed that the proposed multi-task prompting approach yielded a performance boost in a few-shot learning setting.

In a similar study, Simmering and Huoviala^44^ assessed the performance of GPT-3.5 in zero-shot and fine-tuned settings on the ABSA task. They found that fine-tuned GPT-3.5 achieves a state-of-the-art F1 score of 83.8% on both aspect term extraction and sentiment polarity classification of the SemEval-2014 Task 4, improving upon the state-of-the-art model InstructABSA^45^ by 5.7%. However, the performance came at the cost of 1000 times more model parameters to fine-tune, with the associated costs, and an increased latency at inference time. Simmering and Huoviala’s results indicated that while detailed prompts improve performance in zero-shot and few-shot settings, they are not necessary for fine-tuned models.

Zhang et al.^46^ compared the capabilities of LLMs with small LMs trained on domain-specific datasets, on tasks such as conversational classification and multifaceted analysis of subjective texts. Overall, Zhang et al. evaluated performance across 13 tasks on 26 datasets and found that, while LLMs demonstrated satisfactory performance in simpler tasks, they were outperformed in more complex tasks by small LMs where deeper understanding or structured sentiment information is required. Nonetheless, LLMs significantly outperformed smaller models in few-shot learning settings, suggesting their potential when data curation and labelling are limited.

### Semantic gap

The semantic gap—the difficulty to articulate information needs in a way reliably understandable by a computer—is a fundamental challenge in every information retrieval system^47^. CAs are increasingly being used to bridge this gap by allowing users to formulate their queries in natural language^27,39–41^.

In the context of GUI-related CAs, Todi et al.^27^ elicited over one thousand query intents that were manually labelled into different categories and were used to develop a CA prototype. While they presented how intelligent systems can be designed to interact with GUI datasets intuitively, their CA prototype was based on the popular Rasa framework [https://rasa.com] which relies on predefined handwritten rules and user stories. While rule-based approach is highly interpretable and adaptable to new domains and languages, it does not fully capture the variability of natural language and depends on the quality and coverage of the rules, which is clearly not scalable. In order to address this constraint, in our work we employ pre-trained LLMs that offer great flexibility, which can be adapted to new tasks with little programming effort, and can be deployed on commodity mobile devices.

### Transfer learning

Transfer learning is an ML method where a pre-trained model can be used as the starting point for a model on a new task or domain^48^. For example, a model trained on a general-purpose image dataset such as ImageNet^49^ can be adapted to understand more specific images such as X-ray images. Similarly, a model trained on a language disambiguation task can be repurposed for another task such as query disambiguation^50^. One of the main advantages of transfer learning is that a better performance can be achieved as compared to training with only a small amount of data from scratch. This is possible thanks to the adaptation (a.k.a *fine-tuning*) of the model hyperparameters on new data, which allows for rapid and more adequate optimisation. The intuition of fine-tuning in NLP is that, during the pre-training phase, the model has learned rich representations of a language, which enables it to more easily learn (or ‘be fine-tuned to’) the requirements of a downstream language understanding task^51^ such as sentence classification. Interestingly, previous research has found that a well fine-tuned small language model can outperform large-scale ones^52,53^.

Pre-training of LLMs on diverse corpora of unlabelled text has led to several breakthroughs in the use of ML for NLP tasks^54^. Some of the most notable examples of such models include, BERT^55^, RoBERTa^56^, XLNet^57^, PaLM^58^, LLaMA^53^, and the GPT family^59–61^, including its recent and notably popular variant, ChatGPT^62^ and the open-source alternative BLOOM^63^. The main component for the success of these LLMs is the transformer architecture^64^. In this paper, as hinted before, we study a series of lightweight LLMs that can be deployed on commodity mobile devices in order to run inference tasks offline. Figure 1 and Table 2 provide an overview of these LLMs, together with the above-mentioned popular LLMs for comparison.

### Privacy and sustainability issues of LLMs

As LLMs are becoming increasingly more ubiquitous, their impact on users’ privacy becomes more evident. Previous research indicated that LLMs can be susceptible to training data leakage, where sensitive information can be extracted from the models^34^. Due to high number of parameters and size of datasets that are processed during training, large-scale models are especially prone to unintentional memorisation of portions of their training data that could be regurgitated during usage^65^. In turn, CAs built on these models can be vulnerable to such privacy breaches^66^. However, as shown in a recent study, using smaller models can help to mitigate the LLM memorisation issue^67^.

Moreover, human-like interaction offered by present-day CAs opens up possibilities for user nudging, deception, and manipulation^68^. For example, users may disclose more information and/or excessively rely on a personalised agent when they confuse it with a human being^69^. Interestingly, people tend to perceive information sharing practices of a CA (e.g., sharing user’s data with third parties) less negatively if the CA is more socially interactive, and are more likely to make intimate, privacy-sensitive disclosures to such agents^70^. Results of a recent survey on smartphone usage indicate that users vary in their attitudes towards privacy based on personality traits—while some groups are risk-cautious, others are negligent regarding potential threats^71^ which may increase their likelihood of unwittingly compromising their sensitive data. Further, users’ propensity to disclose private information to CAs, combined with the lack of knowledge regarding information collection, the storage and disclosure practices^72^ seem to be at odds with their proclaimed need for transparency and control over their personal data^73^.

Training CAs based on large neural networks is associated with high energy consumption which, in turn, can have a long-term impact on the environment as highlighted in previous work^74,75^. Roller et al.^76^ mentioned that local, on-device, deployment of fine-tuned LLMs can offer a way to enhance privacy and reduce their environmental footprint. Interestingly, Huggins et al.^77^ demonstrated that only 25 training examples are required to achieve a high intent recognition accuracy with a fine-tuned BERT model, showing feasibility of training small language models on a personal laptop. In this paper, we systematically analyse 8 lightweight LLMs in terms of size, performance, and overall fine-tuning time. We also discuss their suitability for deployment on mobile and ubiquitous devices; i.e., LLMs that can be loaded on device and run without the need to communicate with external servers.

## Materials

We used the “Conversations with GUIs” dataset^27^, which comprises of 1317 labelled user queries as a training material for our LLMs. The dataset has elicited example queries at four target variables (a.k.a *intents*: query score, query purpose, response format, and information feature) from three different user groups (end-users, designers, and developers) that were provided together with different GUI screenshots.

Our motivation for choosing this dataset is three-fold. First, it contains out-of-domain data for LLMs and hence model fine-tuning is expected for them to perform adequately. Second, it includes personalised user data that can be considered privacy-sensitive. Third, contrary to medical records datasets, it is publicly accessible. Overall, the dataset provides us with an interesting foundation to explore the trade-off between CAs performance and privacy considerations, which is essential to mobile and ubiquitous systems.

Sample queries for each user group are presented in Table 1. For example, the query “*Show app rating*” (id.227) is an example of an App-level intent whose goal is to obtain numeric information regarding app’s metadata, while the query “*Is there a similar app*” (sic, id.1154) refers to the dataset and its purpose is to filter information. Note that the query types differ in terms of difficulty and some of them were ambiguously labelled, resulting in different types of classification errors that we discuss in ‘Misclassification examples’ section. Also note that, as hinted earlier, even though this dataset was not meant to account for privacy-sensitive data, many queries can be considered as such (see e.g., id.957, id.792 and id.887).

## Methods

In the following, we define our four intent prediction tasks, according to the ground-truth labels provided by the “Conversations with GUIs” dataset, and the chosen models to conduct the tasks. We frame the *query intent prediction* task as classifying a user utterance (or query) under four different categories, which we will refer to as our target variables (or intents):

1. *Query scope (3 classes)* Whether a query refers to an individual GUI, an application, or the entire dataset.
2. *Query purpose (6 classes)* The actionable goal behind the query; e.g. to filter based on some criteria, get more information, request suggestions, etc.
3. *Response format (4 classes)* The expected delivery format of the retrieved information: image, text, numeric, or binary.
4. *Information feature (13 classes)* Particular features that the query was referring to; e.g., related to the accessibility or privacy of an application, its design, etc.

**Table 1:** Query examples (verbatim, in no particular order, chosen at random) for different user groups. We provide an id for each query that refers to the row number in the “Conversations with GUIs” dataset.

Group	(id.) Example query	Scope	Purpose	Format	Info. feat.
Designers	79. *Is there video support?*	UI	Inform	Binary	Functionality
	862. *How can I make a profile?*	UI	Educate	Textual	Settings
	957. *Show me security features*	Dataset	Filter	Image	Privacy
Developers	227. *Show app rating*	App	Inform	Numeric	Metadata
	854. *where is the banner visible?*	UI	Find	Image	Element
	1154. *Is there a similar app*	Dataset	Filter	Binary	Metadata
End-users	16. *How can I save a search?*	UI	Educate	Image	Functionality
	792. *Can i see my credit balance?*	UI	Inform	Binary	Element
	887. *show me all apps using my location!*	Dataset	Filter	Image	Sensor

**Figure 2:**
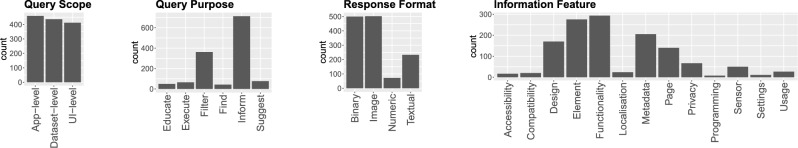
Class distribution of the considered target variables in our study.

Finally, Fig. 2 provides the distribution of classes per target variable. As can be observed, we tackle four multi-class classification problems in this paper. Also, we can see that many of the classes are imbalanced. Therefore, as explained later, we will factor in this observation when measuring intent classification performance.

### Models

We tapped into the LLMs from the Ernie repository to conduct our study: https://github.com/labteral/ernie. The main reason for choosing Ernie is that it is publicly available and contains state-of-the-art lightweight LLMs suitable for on-device deployment. According to a recent survey^78^, the RAM capacity of low-end (< $ 150 ) to mid-end (< $ 550) mobile devices falls in the range between 3 and 8 Gb. Considering that most of the RAM will be occupied by background services and other running apps, we set 2 Gb as the upper-bound for LLM size so that it can be deployed on commodity mobile devices.

*BERT* (Bidirectional Encoder Representations from Transformers)^55^ is a Transformer-based model and the first-of-its-kind groundbreaking LLM. It combines left-to-right and right-to-left training together with a Masked Learning strategy, in which each word in a training sequence is replaced with a special token that the model has to predict. BERT was pre-trained on BookCorpus^79^, that consists of over 11k unpublished books, and on the English Wikipedia.

*RoBERTa* (Robustly Optimized BERT Approach)^56^ builds on BERT’s language masking strategy, where the model learns to predict intentionally hidden sections of text within otherwise unannotated language examples. RoBERTa was trained on the reunion of five datasets: (1) BookCorpus^79^, (2) English Wikipedia, (3) CC-News^80^ which contains 63 million English news articles, (4) OpenWebText^60^, and (5) Stories^81^ which contains a subset of the CommonCrawl corpus^82^ filtered to match the story-like style of Winograd Schemas^83^.

*ALBERT* (A Lite BERT)^84^ is a Transformer architecture based on BERT, but it includes substantially less hyperparameters (10M vs 110M). To accomplish this goal, ALBERT shares same weights across different layers: it has one encoder layer that is applied twelve times on the input. Since ALBERT has about 10 times less hyperparameters than BERT, it puts significantly less strain on computational resources. ALBERT was pre-trained on the same data as BERT.

*XLNet*^57^ is an autoregressive pre-trained LLM that uses bidirectional contexts and maximizes the expected likelihood of a text sentence over all permutation orders, outperforming BERT on 20 different tasks. It incorporates ideas from the Transformer-XL architecture^85^ and overcomes the fixed-length context limitation of BERT and derivative models, resulting in a powerful tool for NLP applications. XLNet was pre-trained on the same datasets as BERT plus CommonCrawl, Giga5^86^ (16 Gb of text), and ClueWeb 2012-B^87^.

**Table 2:** Description of the studied pre-trained lightweight LLMs. Model size is proportional to the number of *Layers*, *Attention Heads*, and trainable *Parameters*.

Model	Layers	Att. heads	Parameters	Size
BERT Base	12	12	110M	440 Mb
BERT Large	24	16	340M	1.2 Gb
ALBERT Base	12	12	11M	63 Mb
ALBERT Large	24	16	17M	87 Mb
RoBERTa Base	12	12	82M	499 Mb
RoBERTa Large	24	16	355M	1.6 Gb
XLNet Base	12	12	110M	565 Mb
XLNet Large	24	16	340M	1.57 Gb

Two remarks are worth mentioning. First, all models can be distinguished based on their size (e.g., Base vs Large) but they all comply with our established 2 Gb limit. Second, all models are case-sensitive, which means that they can disambiguate between common nouns and proper nouns; e.g., an apple (fruit) versus Apple (brand name). This is a convenient feature for any modern CA to be usable in practice.

In addition to these models, we also considered **ChatGPT**, a state-of-the-art proprietary LLM by OpenAI trained on an undisclosed vast amount of data; cf. https://help.openai.com/en/articles/6783457. Our aim was to better understand how lightweight LLMs would compare against the most popular LLM at present. We used the gpt-3.5-turbo-1106 version of ChatGPT, which is available through a paid JSON-based API for custom fine-tuning.

### Fine-tuning procedure

We randomly split all the coded queries in the dataset into three partitions: 70% for training, 10% for validation, and 20% for testing. We use stratified sampling to ensure the same distribution of classes in each partition. The training and validation partitions are used for model fine-tuning, whereas the testing partition is held out for model performance evaluation, as this partition simulates unseen data.

We apply fine-tuning for an incremental number of epochs, from 1 to 20, using a batch size of 16 queries during training and 32 queries for validation. We employ the Adam optimiser^88^ with learning rate $\eta =2^{-5}$ and exponential decays $\beta _1 = \beta _2 = 0.9$. Finally, to stabilise training, we set a clipnorm value of 1. In total, we conducted 720 fine-tuning experiments on the “Conversations with GUIs” dataset, corresponding to the combination of 9 models $\times$ 20 epochs $\times$ 4 query intent prediction tasks.

All experiments, including the additional ones that we report in the Supplementary Materials, were performed in a single Tesla V100 (SXM2, 32 GB) GPU card. Note that, after fine-tuning, the models are ready to be deployed on commodity mobile devices. To ease replication and further follow-up work, we will share our code and model checkpoints upon publication. Please see the Supplementary Materials for details about ChatGPT’s fine-tuning procedure and experiments on other datasets.

## Results

In the following plots we report performance results in terms of Balanced Accuracy and Area Under the ROC curve, as defined below. The dashed horizontal lines denote the classification performance of a random classifier (computed as 100/*c*, where *c* is the number of classes to predict in each case). The random classifier provides a theoretical lower bound, i.e. no LLM should perform worse than random after model fine-tuning. As we can see in the following plots, in all cases ChatGPT achieved the best zero-shot performance, but it was outperformed by other models after fine-tuning.

### Balanced accuracy

**Figure 3:**
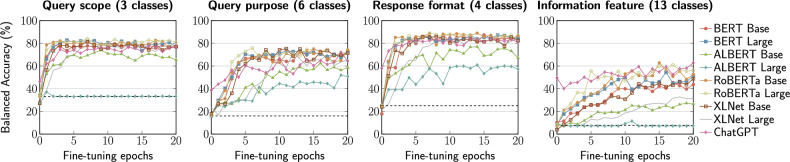
Balanced accuracy results.

Classification accuracy (defined as the number of correct predictions across all predictions) is the standard evaluation metric in classification problems, however it is very sensitive to imbalanced data, i.e., when one of the target classes appears more often than the others; see Fig. 2. Therefore, to account for this, we report Balanced Accuracy instead, which is the arithmetic mean of sensitivity (true-positive rate) and specificity (true-negative rate). Figure 3 summarizes the results.

In terms of **query scope** prediction, ALBERT models are clearly outperformed by all other models. Overall, RoBERTa Large is the best performer, with a balanced accuracy of 84% that is reached after 7 epochs. Notably, BERT Large achieves only slightly worse performance (83%) in just 4 epochs. As for **query purpose**, we can see that all models perform slightly worse than in case of query scope. Specifically, RoBERTa Large reaches the best result of 76% Balanced Accuracy at 6 epochs, however, its performance drops after a few more epochs. The performance degradation of all models in this task is likely explained by the fact that there are as twice as many target variables in this case (6 classes) and thus there may be more room for ambiguity than in the query scope case (3 classes).

Regarding **response format** prediction, this task involves 4 classes and leads to a similar model performance as in the case of query scope prediction. RoBERTa Base is the best performing model, with 89.9% Balanced Accuracy reached after 7 epochs, closely followed by BERT Large that reached the same result in 18 epochs. Overall, the behaviour of all models in this tasks bears a close resemblance to that of the query scope prediction experiments, with the exception of ALBERT Large that performs notably better in this case.

Finally, when it comes **information feature** prediction, the task seems to be the most challenging due to a substantially large number of target variables (13 classes). As can be observed, all models require way more training epochs to achieve their optimal performance. The best performing model for this task is RoBERTa Large with 62.8% Balanced Accuracy in 15 epochs, followed by ChatGPT, which achieved the same performance but after 20 epochs. It is worth pointing out that the performance of most models could have continued improving beyond 20 epochs.

### AUC ROC

**Figure 4:**
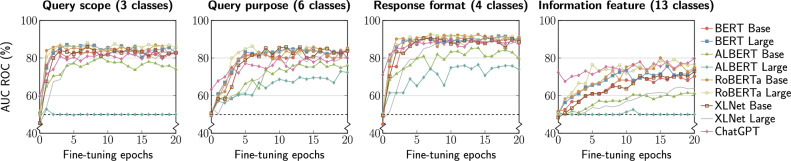
AUC ROC results.

The Area Under the ROC curve (AUC ROC) is a popular metric to assess the discriminative power of any classifier^89^. The ROC curve provides information regarding a model’s false-positive rate against its true-positive rate, across a range of classification thresholds, and the AUC ROC is the area under such a curve. Since AUC ROC is defined for binary classification problems and all our experiments have more than two classes, we compute it in a one-vs-all fashion, to account for multi-class classification. Figure 4 summarizes the results.

As can be seen, there is an analogous trend to the results observed in the Balance Accuracy experiments (cf. Fig. 3). In terms of **query scope** prediction, the best performance is achieved by the RoBERTa models, with models reaching 87% AUC ROC in 3 epochs, while the ALBERT models performed the worst. As for **query purpose**, the best performance is exhibited by RoBERTa Large, which reaches 86% AUC ROC in 6 epochs. Again, ALBERT models performed the worst. This is likely explained due to its substantially smaller number of hyperparameters as compared with the other models, making ALBERT unsuitable for fine-tuning to GUI-related tasks.

Regarding **response format** prediction, RoBERTa Large is the best performing model, reaching 93% AUC ROC in 7 epochs. ALBERT Large performed notably better (76% at 12 epochs) when compared to its performance for the other target variables, where it did not achieve any improvement.

Finally, when it comes **information feature** prediction, RoBERTa Base converged the fastest and reached the best AUC ROC of 80% at 15 epochs. ChatGPT achieved its peak performance of 79.8% at 20 epochs. On the other hand, contrary to Balanced Accuracy, where there were larger discrepancies between XLNet Base and XLNet Large models, this time XLNet Base tended to perform better than its Large variant.

### Summary of findings

We have seen a clear and interesting relationship between the number of classes and classification performance of all the studied LLMs. First, for a small number of classes the trend resembles a logarithmic curve with models saturating after just a few epochs. Then, as the number of classes increases, the curve gets flatter and the models take longer to reach optimal performance. While ChatGPT outperformed all other models initially (zero-shot classification), it exhibited the same fine-tuning trends. It was also interesting to notice that it was outperformed by other models after fine-tuning, in line with previous experiments performed by Zhang et al.^46^.

Our analysis underscores the importance of LLM fine-tuning for query intent prediction tasks, and highlights the need to select the appropriate model for the task at hand. Our analysis also helps to determine the optimal model choice for predicting query intent based on the trade-off between model complexity and efficiency. Importantly, we fine-tuned all lightweight LLMs in a commodity GPU card, so researchers and practitioners can easily reproduce our findings.

The general trend we observed is that, over time, the models converged to an optimum or “sweet spot”. We should note that the small fluctuations observed in our performance metrics are attributed to fluctuations in the training loss over epochs. They are mostly due to (1) the stochastic nature of gradient descent, (2) the fact that we cannot fit *all* queries in a single batch, and (3) the large size of the models in proportion to the small size of the dataset.

For the sake of conciseness, Table 3 provides a summary of the top-3 performing models for the query intent prediction tasks considered. All models were ranked on the basis of the optimum value of Balanced Accuracy; i.e., when each model achieved maximum Balanced Accuracy in a minimum number of epochs. The table also reports the time (in minutes) to fine-tune each model until such an optimum state. We can see that RoBERTa Large is the only model that is systematically ranked among the top-3 for all tasks. Please note that, instead of reporting a summary of *all* models, by analysing the top-3 best performing models, we provide more concise and focused insights for researchers and developers interested in prototyping their chatbots. As a matter of fact, in the initial stages of research it may be more practical to experiment with a model that allows for faster iteration cycles. For example, RoBERTa Base provides similar performance than RoBERTa Large, yet it takes half the time to be optimally fine-tuned.

**Table 3:** Summary of top performing models for each task after fine-tuning, based on the achieved balanced accuracy (higher is better) and number of epochs (lower is better). The ‘Zero-shot Acc.’ column denotes classification accuracy before fine-tuning. Fine-tuning times (lower is better) are computed until the best epoch reported in their respective row. ChatGPT only made it to the top-3 for the Information Feature task.

Task	Top-3 models	Zero-shot Acc. (%)	Bal. Acc. (%)	Epochs	Time (min)
Query scope (3 classes)	RoBERTa Large	33.3	84.2	7	6.59
	BERT Large	33.3	82.8	4	3.75
	RoBERTa Base	33.3	82.6	17	7.11
Query purpose (6 classes)	RoBERTa Large	16.6	75.8	6	5.43
	XLNet Base	17.9	75.3	10	4.91
	BERT Base	15.8	75.0	15	6.85
Response format (4 classes)	RoBERTa Base	24.2	89.0	14	5.88
	RoBERTa Large	25.0	88.9	7	6.51
	Bert Base	17.8	87.4	15	6.03
Information feature (13 classes)	RoBERTa Base	7.1	62.8	15	6.41
	ChatGPT	49.4	62.8	20	58.5
	RoBERTa Large	8.1	54.4	16	14.67

To further contextualise our findings, we would like to highlight a longstanding discussion regarding the trade-off between model complexity and the availability of training data. As shown in previous work, having better quality data rather than more data will lead to enhanced model performance, regardless of its complexity^25,26^. This relationship seems to be reflected in our experimental results as well, where larger models (e.g. BERT Large) or models trained on more data sources (e.g. XLNet) did not always lead to a better performance.

## Discussion

We begin by answering our main research questions regarding model performance (**RQ1**) and the relationship between number of training epochs and performance (**RQ2**). We also provide some misclassification examples to better contextualise our findings. We then discuss the implications of our research to relevant stakeholders, consider the limitations of our study, and propose several possible avenues for future work.

### Model performance

Before fine-tuning, all lightweight LLMs were able to capture *general* language features and patterns, but they did not exhibit adequate performance for any of the tasks at hand. After fine-tuning, however, the models learned from the training data and demonstrated much better performance, allowing them to better tackle each of the considered prediction tasks. It should be noted that only ChatGPT performed well at zero-shot classification, outperforming all lightweight LLMs initially, but it was later outperformed by other models after fine-tuning. ChatGPT’s excellent zero-shot performance is attributed to the fact that it is much more complex and has much more knowledge about the (written) world than any of the lightweights LLMs we have studied.

**RQ1a:** *Which pre-trained models achieve the highest balanced accuracy after fine-tuning for predicting query intent in terms of: scope, purpose, response format, and information feature?*

We observed that RoBERTa models perform the best in all of the four prediction tasks. Specifically, RoBERTa Large performed best for query scope (84.2%) and query purpose (75.8%), while RoBERTa Base was the best for response format (89%, outperforming RoBERTA Large by 0.1 points) and information feature (62.8%). It has to be noted however, that both models significantly differed in their fine-tuning time until convergence, as discussed in the next section.

**RQ1b:** *Is there a model that performs best in all of the four tasks above?*

Based on our previous discussion, we posit that while there is no single winner-takes-all model, except ChatGPT which excels at zero-shot classification tasks, RoBERTa Large is the most sensible model for all tasks. Strictly speaking, while RoBERTa Base achieved the best performance in predicting response format, it was by a negligible margin as compared to RoBERTa Large: 89% versus 88.9%, and the differences are not statistically significant. Therefore, for the best overall performance (i.e., highest accuracy), we recommend RoBERTa Large for development of CAs for GUI assistance.

Nonetheless, it should also be noted that despite its small size (almost one third of RoBERTa Large), RoBERTa Base performs exceptionally well in the most challenging task of information feature prediction which contains 13 different classes (see Table 3 for performance details). Therefore, it should be considered for disambiguation tasks with large number of classes, especially given its short convergence time (6 min vs 16 min for RoBERTa Large).

### Relationship between training epochs and performance

As hinted previously, each model has its preferred “fine-tuning sweet spot”. In the following, we discuss the variability observed in terms of epochs for each model to achieve their best performance.

**RQ2a:** *What is the minimum number of fine-tuning epochs for each pre-trained model?*

At least six to seven epochs are required to achieve competitive performance in all of the considered prediction tasks, except from information feature prediction, were at least fifteen epochs are usually required. Further, with the exception of information feature, beyond fifteen epochs some models started to overfit. This could be justified by the high complexity of this task which, compared to the other tasks, requires longer training times. Overall, it is advised not to fine-tune these models beyond fifteen epochs on a dataset like the one we have analysed.

**RQ2b:** *What is the optimal number of fine-tuned epochs for each model to achieve the best performance?*

We observed that this number is consistently in the 7–15 range for all tasks, with the exception of the challenging information feature prediction task, where all models needed more time to converge. We observed that all models apart from ALBERT family and XLNet Large required only 3–5 epochs to start approaching their optimum performance. This observation is in line with previous work that reported similar ranges for BERT models^90,91^. When it comes to information feature prediction, for BERT family models, 10–15 epochs were needed to reach optimal performance, while for RoBERTa models this range fell between 7 and 12 epochs. A notable exception here are the XLNet models, whose performance followed a trend that was likely to peak beyond 20 epochs. Overall, we observed that a larger number of classes per intent implies a more gradual learning curve.

### Misclassification examples

Table 4 contains examples of queries misclassified by the top-3 best performing models reported before. While overall the models performed quite well, some of the user queries proved to be difficult due to their ambiguous character. For example, “*How to create a shopping basket?*” (id.985) was predicted by RoBERTa Large as a ‘Suggest’ rather than an ‘Educate’ purpose, which, given the limited context, could theoretically fall into both of these categories. Similarly, in ‘*What data are we collecting*” (id.581), RoBERTa models predicted information feature to be a ‘Metadata’ instead of a ‘Privacy’ class. Again, the query was possibly challenging to the model due to its brevity and lack of more extensive contextual information. Another ambiguous example, which proved problematic to ChatGPT and RoBERTa Large, is “*Where is the privacy?*” (id.813), which was identified as a request for a ‘Binary’ or ‘Textual’ response rather than an ‘Image’ response format. It was again difficult to predict the ground-truth format since both are equally sensible candidates to resolve this query.

It also should be noted that the dataset contains some queries that are duplicated or near-duplicated but have different ground-truth labels. For example, “*Is there a login page?*” (id.21 and id.65) is labeled in terms of Information Feature as ‘Page’ for id.21 and as ‘Functionality’ for id.65. These cases, while were not frequent, may have introduced some noise in the models and thus made the prediction tasks a bit more challenging.

**Table 4:** Examples of classifications errors committed by the top-3 performing models, highlighted in bold. The same query is tested cross-model.

Task	Top-3 models	(id.) Example query	Query intent	
			Predicted	Ground-truth
Query purpose	RoBERTa Large		Educate	Educate
	BERT Large	985. *How to create a shopping basket?*	Educate	Educate
	RoBERTa Base		**Suggest**	Suggest
Query scope	RoBERTa Large		**UI-level**	App-level
	XLNet Base	916. *Can I enlarge the app window?*	**UI-level**	App-level
	BERT Base		**UI-level**	App-level
Response format	RoBERTa Base		Image	Image
	ChatGPT	813. *Where is the privacy?*	**Binary**	Image
	RoBERTa Large		**Textual**	Image
Information feature	RoBERTa Base		**Metadata**	Privacy
	RoBERTa Large	581. *what data are we collecting*	Privacy	Privacy
	BERT Large		Privacy	Privacy

### Implications

Overall, users are currently faced with two alternatives. They can either use ChatGPT without fine-tuning to achieve competitive classification performance (especially for intents having a large number of classes) yet at the expense of compromising their privacy and some monetary costs ($0.008 per 1K tokens, around $1 per intent category in the “Conversations with GUIs” dataset), or fine-tune lightweight LLMs on their own premises to achieve better performance.

In the following, we discuss the implications of our findings to relevant stakeholders, including developers, designers, end-users, and the mobile and ubiquitous multimedia community. It should be noted that these recommendations are mostly based on our findings on the “Conversations with GUIs” dataset, which is more challenging that other NLP datasets. We refer to the Supplementary Materials for additional experiments that highlight superior results for most of the models we have studied in this paper.

#### For developers

Without fine-tuning, all models except ChatGPT perform like a random classifier in most cases (see dashed lines in Figs. 3 and 4). It is clear thus that lightweight LLMs are not ready to support the users’ needs in a CA context without proper fine-tuning. This can be explained by the fact that all the studied lightweight LLMs were pre-trained on general-purpose data, whereas the “Conversations with GUIs” dataset^27^ is specific to user interfaces so it can be considered ‘out-of-domain’ data. Interestingly, right after just one epoch all lightweight LLMs exhibited a boost in their classification performance results. Moreover, we observed that smaller (Base) models do not necessarily require less number of fine-tuning epochs than larger models.

#### For designers and end-users

CAs have potential to streamline interaction with GUIs by offering an additional channel of communication. For example, users can issue conversational queries (via text of voice) to quickly access information regarding an app’s privacy settings (e.g., GPS tracking) that would be otherwise hidden in a long-winded technical specification document. Knowing which model offers the best accuracy to memory footprint trade-off can help users decide if performance gains are worth the additional time spent on interacting with the model. It should be noted that hardware limitations may make fine-tuning of very large models infeasible for users without access to high-performance computing. This point also applies to designers who work for companies that are concerned about economical use of the available assets. Effectively, on a global scale, our work can contribute to more reasonable and greener use of computational resources.

#### For mobile and ubiquitous computing

Ubiquitous applications are expected to operate in dynamic environments, in which mobile devices can operate seamlessly, without relying on data connectivity. Our work sets a cornerstone in this regard by allowing the interested researchers to deploy efficient lightweight LLMs on commodity hardware for CA-based applications. Some examples of these applications include, for example, developing multi-party CAs^92^ or maintaining reading flow in e-readers^93^. Therefore, our work should be seen as an enabling technology for the mobile and ubiquitous computing community.

As highlighted by Mhlanga^94^, protecting data privacy not only is an ethical obligation that demonstrates respect for users’ rights but should also be a priority for company owners and developers that they employ. While the General Data Protection Regulation (GDPR) legislation requires companies and organisation to protect the personal data of end-users, in practice achieving a 100% compliance may be unlikely. In our investigation, we envision that end-users can run CAs based on lightweight LLMs locally on their own device to avoid sending queries to cloud-based services, thus protecting their privacy. Depending on the level of tech-savviness and the available resources (the GPU card we used in our experiments costs around $3k.), lightweight LLMs can be directly trained by end-users themselves or supplied as a one-off purchase chatbot plugin supplied by a company.

### Limitations and future work

It should be noted that while we adopted 2 Gb of RAM as our upper-bound for model deployment, this size may exceed the capacity of some older mobile devices, so it is advised to work with models well below that threshold to ensure a wider range of compatibility. While, overall, larger models yielded best performance in our study, XLNet Base (for query purpose) and RoBERTa Base (for query scope) match the top performing models closely, offering a viable alternative while substantially reducing required RAM ($\sim$60% reduction) for older mobile devices which have lower memory capabilities.

One aspect that we have not explored in this work is the analysis of runtime performance on low- and mid-end devices, as we did not deploy our models. This implementation aspect should be explored in future work. In addition, it is advised to consider an online learning scenario, where new (unseen) queries are provided by end-users as they interact with the models with their devices. This can be implemented following the same fine-tuning methodology that we have presented, but using a batch size of 1, to ingest one new query at a time.

Future work could consider more advanced fine-tuning techniques such as delta tuning^95^ and low-rank adaptation^96^ in order to fine-tune LLMs that are prohibitively costly (in terms of computational resources) to many researchers, such as those depicted in Fig. 1. However, it should be noted that these techniques require much more data to converge compared to traditional fine-tuning^25,97^. Finally, future work should also explore runtime and battery consumption on specific models of low- and mid-end mobile devices to practical insights to provide practical insights regarding deployment of light weight LLMs on commodity devices.

Moving forward, we would like to propose three possible applications of privacy-preserving lightweight LLMs to existing products that can be developed in the future to support different groups of GUI users. Firstly, a CA can support developers who could use it on demand from the command line interface. Alternatively, such a CA can be also embedded in integrated development environments such as Visual Studio Code [https://code.visualstudio.com/]. Secondly, designers can benefit from a CA integrated into interface design tools like Figma [https://www.figma.com/] or Sketch [https://www.sketch.com/] to assist them in collaboratively creating new interfaces. In this context, the CA could aggregate anonymised user queries in an ethical way (e.g. removing brand names or entities using NLP methods) for users who opt-in to improve the CA functionality by informing a third party service. Thirdly, since end-users are mostly concerned about privacy features^27^, we suggest that CAs could be integrated in Google Play [https://play.google.com/store/games] or the iOS App Store [https://www.apple.com/app-store/] so that users can query privacy and other metadata related features of specific applications.

To conclude this section, we would like to acknowledge that, despite its privacy-enhancing potential, fine-tuning LLMs on our own premises can raise some ethical issues. Since there is no oversight regarding how the models will be deployed “in the wild”, they could potentially be applied to malicious activities such as stealing user credentials (cf. FraudGPT [https://thehackernews.com/2023/07/new-ai-tool-fraudgpt-emerges-tailored.html], WormGPT [https://www.infosecurity-magazine.com/news/wormgpt-fake-emails-bec-attacks/], and the like). Nonetheless, we believe that, all things considered, fine-tuning lightweight LLMs on premise brings more benefits to users than risks.

## Conclusion

We have studied how to best fine-tune different lightweight pre-trained LLMs for on-device query intent prediction to support users during GUI-related interactions with CAs. Our results indicate that there exists a middle ground between giving away our privacy to some third party cloud service in exchange for boosted performance (specially in zero-shot classification scenarios) and resorting to traditional CA developments that do not scale well. While RoBERTa Large was shown to be the best performer overall, among all the models explored, RoBERTa Base and XLNet Base offered the best trade-off between performance (intent prediction accuracy and AUC ROC) and memory footprint, and thus they may be equally suitable for on-device CA deployment. Taken together, our findings provide valuable insights for different stakeholders who use or work with GUIs, and who are interested in developing mobile and ubiquitous systems that need to balance performance and memory footprint while also considering privacy implications. Our model checkpoints and software are publicly available at https://luis.leiva.name/llmgui/.

### Supplementary Information

Supplementary Information 1.

## Data Availability

The datasets used and/or analysed during the current study are available from the corresponding author on reasonable request.
