# Author Correction: On-device query intent prediction with lightweight LLMs to support ubiquitous conversations

**DOI:** 10.1038/s41598-024-66714-6

**Published:** 2024-07-11

**Authors:** Mateusz Dubiel, Yasmine Barghouti, Kristina Kudryavtseva, Luis A. Leiva

**Affiliations:** University of Luxembourg, 4365 Esch‑sur‑Alzette, Luxembourg

Correction to: *Scientific Reports* 10.1038/s41598-024-63380-6, published online 03 June 2024

In the original version of this Article, the corresponding URL for the ‘model checkpoints and software’ was incorrect.

“Our model checkpoints and software are publicly available at [URL TBA].”

now reads,

“Our model checkpoints and software are publicly available at https://luis.leiva.name/llmgui/.”

The original Article has been corrected.

