# Supplementary material for: On-device query intent prediction with lightweight LLMs to support ubiquitous conversations
Source: Sci Rep. 2024 Jun 3;14:12731. doi: 10.1038/s41598-024-63380-6 (PMC11148047; doi:10.1038/s41598-024-63380-6)
Supplement: Supplementary file 1 — Supplementary Information 1. [file 41598_2024_63380_MOESM1_ESM.pdf]

Supplementary Materials of ‘On-device Query Intent Prediction with Lightweight LLMs to Support Ubiquitous Conversations’

Mateusz Dubiel[1,+], Yasmine Barghouti[1], Kristina Kudryavtseva[1], and Luis A. Leiva[1,+,*]

[1]University of Luxembourg, L-4365 Esch-sur-Alzette, Luxembourg
[*]Corresponding author: luis.leiva@uni.lu
[+]These authors contributed equally to this work.

ABSTRACT

This document provides information about ChatGPT fine-tuning and additional experiments on other datasets.

ChatGPT fine-tuning

We chose the `gpt-3.5-turbo-1106` model version because this is the one currently used in the free version of ChatGPT. This model is specifically designed for “instruction following”, meaning that the model is able to produce concise outputs for specific instruction-like requests (as compared to longer conversational style replies). The OpenAI API allows to specify only 3 hyperparamters for fine-tuning: `n_epochs` (which we iteratively increased from 1 to 20), `batch_size` (we used a value of 64), and `learning_rate_multiplier` (we used 2),

The training data was formatted as a JSONL file to be sent to the fine-tuning endpoints, where every line in that JSONL file consists of a dictionary with three entries: system content (contextual prompt), user content (query), and assistant content (query label). We used the following contextual prompt template: *“Please determine the `{intent}` of the given query. Possible values are listed here: `{labels}`. Please respond only with one values from the list, no other text.”* where `{intent}` is one of the four intent names (e.g. query scope) and `{labels}` is the list of possible intent labels (e.g. for the ‘query scope’ intent, the list is {App-level, Dataset-level, UI-level}). An instantiated prompt example for the ‘response format’ intent is the following one: *“Please determine the response format of the given query. Possible values are listed here: ["Binary", "Image", "Numeric", "Textual"]. Please respond only with one value from the list, no other text.”* According to recent prompt guidelines[1], we tried to introduce ChatGPT itself in the prompts with a concrete role (e.g. “You are an assistant that helps users to answer their queries” or “You are a query itent classifier”) but unfortunately this did not improve performance.

The remainder of this fine-tuning procedure is exactly the same as the one we followed for the lightweight LLMs; i.e., iterative fine-tuning up to 20 epochs over the training data and subsequent evaluation over the testing data.

Additional datasets

We replicate our fine-tuning methodology on 4 additional (public) datasets, depicted in Table 1. These datasets are well-established benchmarks for query classification experiments and conversational question answering systems. The “chatbot style” column indicates whether queries are very short, unstructured, and command-like[2]. The “constrained data” column indicates that the amount of training data is rather small[2].

Both TREC datasets below were compiled by Li and Roth[3]. In TREC-6 each query is labeled according to one of 6 possible intents (e.g. ‘entity’, ‘location’, ‘number’), whereas in TREC-50 the number of possible intents is 50 (e.g. ‘entity:animal’, ‘entity:event’, ‘location:country’, ‘location:other’). The Chatbot and AskUbuntu datasets were compiled by Braun et al.[4]. Queries in the Chatbot dataset are labeled according to 2 intents (either ‘FindConnection’ or ‘DepartureTime’), whereas in the AskUbuntu dataset, queries are labeled according to 5 intents (e.g. ‘Setup Printer’, ‘Software Recommendation’, ‘Shutdown Computer’).

We report in the following plots the results of fine-tuning all the model considered, both in terms of balanced accuracy (Figure 1) and AUC ROC (Figure 2). We can observe very similar trends as those shown in the plots of the “Conversations with GUIs” dataset, namely: (i) excellent zero-shot performance of ChatGPT, but outperformed by other models after fine-tuning; (ii) stabilization to a “sweet spot” overall; and (iii) a quick (logarithmic) convergence to such as sweet spot after 3–5 epochs when the number of intent classes is small (otherwise the convergence happens rather slowly). RoBERTa models were again the best performers overall. We also observed the same poor performance of ALBERT models, in particular ALBERT Large.

Dataset	Intents	Queries	Chatbot style	Constrained data
TREC-6	6	5952	No	No
TREC-50	50	5952	No	No
Chatbot	2	206	Yes	Yes
AskUbuntu	5	162	No	Yes

Supplementary Table 1. Overview of the additional datasets.

Therefore, we can conclude that, all in all, it is beneficial to do on-premise fine-tuning of lighweight LLMs, considering the privacy implications of accessing larger models via clouds services or web APIs.

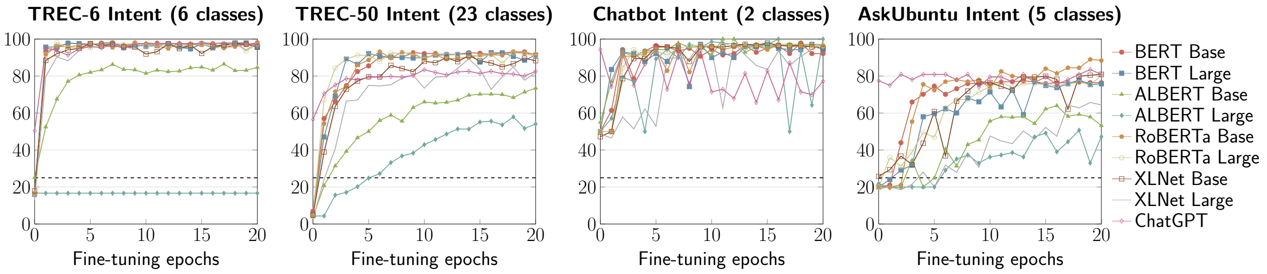

Supplementary Figure 1. Balanced accuracy results on additional datasets.

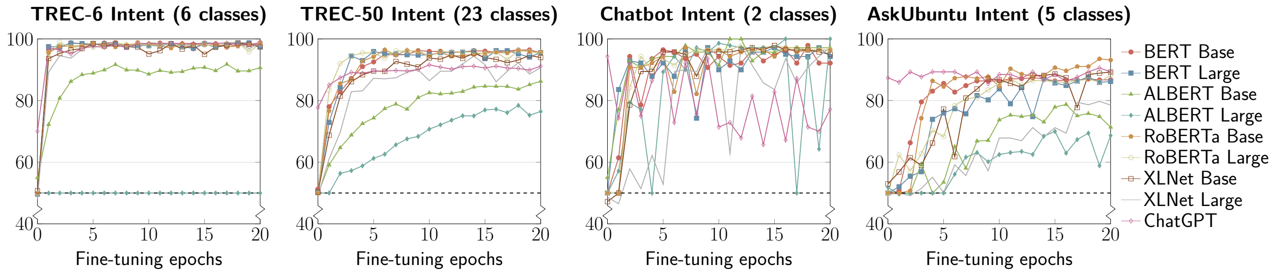

Supplementary Figure 2. AUC ROC results on additional datasets.

References

1. Khurana, A., Subramonyam, H. & Chilana, P. K. Why and when LLM-based assistants can go wrong: Investigating the effectiveness of prompt-based interactions for software help-seeking. In *Proc. IUI* (2024).

2. Larson, S. *et al.* An evaluation dataset for intent classification and out-of-scope prediction. In *Proc. EMNLP-IJCNLP* (2019).

3. Li, X. & Roth, D. Learning question classifiers. In *Proc. COLING* (2002).

4. Braun, D., Hernandez-Mendez, A., Matthes, F. & Langen, M. Evaluating natural language understanding services for conversational question answering systems. In *Proc. SIGDIAL* (2017).
